# Occupational health responses to COVID‐19: What lessons can we learn from SARS?

**DOI:** 10.1002/1348-9585.12128

**Published:** 2020-05-13

**Authors:** David Koh, Hui Poh Goh

**Affiliations:** ^1^ PAPRSB Institute of Health Sciences Universiti Brunei Darussalam Gadong Brunei Darussalam; ^2^ SSH School of Public Health National University of Singapore Singapore

**Keywords:** coronavirus, COVID‐19, health care, occupational health, outbreaks, public health, SARS‐CoV‐2

## Abstract

On 31 December 2019, the World Health Organization (WHO) received reports of pneumonia cases of unknown etiology in the city of Wuhan in Hubei Province, China. The agent responsible was subsequently identified as a coronavirus—SARS‐CoV‐2. The WHO declared this disease as a Public Health Emergency of International Concern at the end of January 2020.

This event evoked a sense of déjà vu, as it has many similarities to the outbreak of severe acute respiratory syndrome (SARS) of 2002‐2003. Both illnesses were caused by a zoonotic novel coronavirus, both originated during winter in China and both spread rapidly all over the world. However, the case‐fatality rate of SARS (9.6%) is higher than that of COVID‐19 (<4%). Another zoonotic novel coronavirus, MERS‐CoV, was responsible for the Middle East respiratory syndrome, which had a case‐fatality rate of 34%.

Our experiences in coping with the previous coronavirus outbreaks have better equipped us to face the challenges posed by COVID‐19, especially in the health care setting. Among the insights gained from the past outbreaks were: outbreaks caused by viruses are hazardous to healthcare workers; the impact of the disease extends beyond the infection; general principles of prevention and control are effective in containing the disease; the disease poses both a public health as well as an occupational health threat; and emerging infectious diseases pose a continuing threat to the world. Given the perspectives gained and lessons learnt from these past events, we should be better prepared to face the current COVID‐19 outbreak.

On 31 December 2019, the World Health Organization (WHO) was informed of pneumonia cases of unknown etiology detected in Wuhan city in Hubei Province, central China. By 9 January 2020, WHO released a statement on the cluster of cases, which stated that “Chinese authorities have made a preliminary determination of a novel coronavirus, identified in a hospitalized person with pneumonia in Wuhan”.^1^ The virus was initially referred to as 2019‐nCoV, but has since been re‐named as SARS‐CoV‐2 on 12 February 2020. The WHO declared this disease, COVID‐19 as a Public Health Emergency of International Concern at the end of January 2020.^2^ In a span of less than 2 months, there have been over 70,000 confirmed cases and over 1800 deaths due to the infection. By early April 2020, there were over 1.1 million confirmed cases and over 62 000 deaths spread over 200 countries and territories.^3^

Initial reports suggested that the overall case‐fatality of COVID‐19 infection appeared to be approximately 2%.^4^ The case‐fatality was much higher in the city of Wuhan (currently around 4.9%) as compared to other parts of China and the rest of the world. The disease has an estimated mean incubation period of 5.2 days (95% CI 4.1‐7.0) and a basic reproductive number (Ro) of 2.2 (95% CI 1.4‐3.9).^5^ It is possible that people with COVID‐19 may be infectious even before showing significant symptoms.^6^ However, it is believed that those who have symptoms are the ones who are primarily causing the spread of the infection. These figures have been updated recently, with an estimated Ro of between 2.0 and 2.5 for SARS‐CoV‐2, incubation period of 1‐14 days (mean 5‐6) days and a mortality rate of about 3.8%, based on a larger sample of 55 924 cases.^7^

This event evoked a strong sense of déjà vu, as it has many parallels to the outbreak of SARS during 2002 and 2003. Similar to COVID‐19, SARS originated during winter in China, spread rapidly all over the world affecting 37 countries, and was caused by a zoonotic novel coronavirus. However, in comparison, SARS affected far fewer people (8098 reported cases) but had a higher case‐fatality rate of 9.6% (774 deaths). Another zoonotic novel coronavirus, MERS‐CoV, originated in the Middle East in 2012. In all, there have been 2494 reported MERS‐CoV cases resulting in 858 deaths (case‐fatality rate, 34%) in 27 countries. Outbreaks of MERS have been reported in hospitals in countries such as Saudi Arabia, Jordan, and South Korea.^8^

Our experiences in coping with the previous SARS and MERS outbreaks have better prepared us to face the new challenge posed by COVID‐19. In particular, we learnt many valuable lessons from dealing with the SARS outbreak, which was an unprecedented event. The perspectives and experiences gained from managing SARS,^9^ and comparisons to our responses to the COVID‐19 outbreak include the following:

## 1 RECOGNITION THAT WORKING IN HEALTH CARE SETTINGS CAN BE HAZARDOUS TO HEALTH

Healthcare workers (HCWs) in health care establishments include doctors, nurses, laboratory and paramedical staff, health attendants and cleaners. Other than health care workers, anyone who are physically present or associated with health care institutions were at high risk of infection by SARS‐CoV. Worldwide, HCWs comprised a significant 21% of all SARS patients, but in countries such as Canada and Singapore, more than 40% of the patients were HCWs.^10^ Performance of certain procedures, such as intubation and nebulization of SARS patients was recognized as having a significant risk of infection. However, even low‐exposure situations and transient exposures to infected cases posed infection risks. There were also reports of “super‐spreaders,” who often were initially undiagnosed, and who spread the disease to clusters of HCWs.

Healthcare workers are also a recognized high‐risk exposure group to SARS‐CoV‐2. As of 2 March 2020, more than 3400 HCWs have been infected in China, with 13 deaths reported.^11^ A COVID‐19 “super‐spreader” was reported in a Wuhan hospital. The patient presented with abdominal symptoms and was initially admitted to a surgery department, resulting in over 10 HCWs being infected.^12^ In many other countries, currently thousands of HCWs have been infected, and hundreds have died, though not all have occurred because of occupational exposure. This is because household and community transmission have also played a role in the infection of HCWs.

In general, HCWs are now better equipped and better trained and prepared with infection control techniques as compared to the time of SARS. The number of HCWs as a proportion of all cases of COVID‐19 appears to be smaller and HCWs comprise less than 20% of cases. Several factors may have contributed to this. For example, in Singapore, there are established occupational medicine departments to protect HCWs in major Singapore government hospitals in 2020. In comparison, no such departments existed in 2003. To reduce the risk of occupational exposure to infection, personal protective equipment (PPE) have been stockpiled, HCWs are mask fitted, repeated training in infection control techniques have been given, and strict adherence to infection control protocols is mandated. There is also extensive daily monitoring of health among staff.^13^

World Health Organization has developed several technical guidance documents regarding COVID‐19 for HCWs, including rights, roles and responsibilities of HCWs^14^ which comprises key considerations for safety and health. As part of a WHO preparedness and response initiative, they have also established a risk assessment tool that is to be used by health care facilities to determine the risk of SARS‐CoV‐2 infection of all HCWs who have been exposed to a COVID‐19 patient. This tool also provides recommendations for appropriate management of these HCWs, according to their infection risk.^15^

However, the magnitude of the pandemic, with its explosive increase in number of infected patients requiring treatment in health care facilities, has resulted in health care establishments being overwhelmed in many countries. There are shortages of PPE such as N95 masks and surgical masks for health care workers, and ventilators for patients even in the more developed countries with robust health care systems.^16^ There has also been a profound loss of trust in authority with perceptions that policy is guided more by scarcity than science.^17^ In the developing countries, the situation is even more dire.

## 2 THE IMPACT OF THE DISEASE EXTENDS BEYOND THE INFECTION

During the SARS outbreak, HCWs in affected countries worked under great stress and in constant fear. Besides being exposed to the virus, they experienced fatigue, burnout, stigma and were at risk for physical and psychological violence. The need to protect themselves by having to wear uncomfortable PPE at all times, the necessity to monitor body temperature several times a day, enforced restriction of movements within and between health care establishments and having to work long hours in physically separate teams were common features of health care work.

About a third (29%‐35%) of hospital workers in Toronto^18^ experienced a high degree of distress, as measured on the Impact of Event Scale. In Singapore, over ten thousand HCWs in nine health care settings were surveyed during the SARS outbreak.^19^ Many reported feeling more stressed at work, experiencing an increase in workload and having to work overtime. Most respondents agreed that “people close to me are worried for my health,” and that “people close to me are worried they might get infected through me.” In addition, there was also fear and stigmatization of HCWs and their family members from the public because of their occupation. (Table 1).

**TABLE 1:** Responses of Healthcare workers in Singapore during the SARS outbreak, 2003 (n = 10 511)

I feel more stressed at work	56%
I experienced an increase in workload	53%
I have to perform work that I normally don't do	54%
I had to work overtime	36%
People close to me are worried for my health	87%
People close to me are worried they might get infected through me	69%
People avoid me because of my job	49%
People avoid my family members because of my job	31%

Note

Source: Koh et al. (2005)^19^.

During this COVID‐19 outbreak, HCWs are similarly working under extreme conditions over long hours. Many HCWs have fears for their personal health and many have their family members worried for them. The need for management of stress and fatigue among HCWs is important and should be recognized and provided for.^20^

At the same time, stigmatization and ostracization of HCWs have been witnessed. Due to their occupation, HCWs are shunned and harassed by some members of a fearful public. These reactions arise largely from ignorance and anxiety. Such adverse reactions have also been directed towards other groups of people, such as those under quarantine, or persons of specific races and nationalities. The CDC has identified persons of Asian descent, people who have travelled and emergency responders or healthcare professionals as groups who may be at risk of being stigmatized.^21^

On the other hand, there have are also been positive reactions from the public and strong expressions of gratitude and support for HCWs. Many members of the public do appreciate the HCWs’ dedication to work and the sacrifices they make and there has been a general outpouring of support from the public for HCWs in many countries.

Besides the obvious stressors of working long hours under conditions of risk of infections, and separation from their families and loved ones another mental health challenge faced by health care workers is the dilemma they face when deciding how to ration scarce health care resources to patients.^22^ In countries such as Italy, where the number of patients who need ventilators outnumbered the available equipment, health care workers were forced to make uncomfortable life or death decisions.^23^ The lack of resources contributed in part, to the high COVID‐19 death rate in Italy. This is unfortunately another challenge seen in COVID‐19 outbreak that was not encountered during the SARS outbreak.

Mental health support for HCWs can be provided via multidisciplinary mental health teams, which include psychiatrists, psychiatric nurses, clinical psychologists, and other mental health workers. Regular, accurate and clear communication updates should be provided in order to allay prevailing uncertainty and fear that the HCWs are experiencing. HCWs caring for COVID‐19 patients may also require regular clinical screening for depression and anxiety.^20^

However, specific mental health issues may need to be managed by different approaches. For example, one possible solution to help clinicians directly involved in managing critically ill patients from making ethically difficult choices such as deciding who receives ventilator care, is to form a triage committee.^24^ Such a committee can comprise volunteers, including respected clinicians and leaders, among their peers. The committee can be tasked to make these difficult choices, in order to spare the frontline clinicians from the dilemma of rationing scarce medical resources.

## 3 GENERAL PRINCIPLES OF PREVENTION AND CONTROL ARE EFFECTIVE IN CONTAINING THE DISEASE

Severe acute respiratory syndrome was spread to HCWs mainly by direct mucous membrane contact with infectious respiratory droplets and exposure to contaminated surfaces. Prevention and control measures were early detection and isolation of cases and quarantine of exposed members of the public. These measures were effective. For example, secondary cases of SARS were minimal when the source cases were isolated within 2 days of onset of symptoms. However, if isolation was delayed, the number of secondary cases increased rapidly.

For HCWs, effective preventive measures involve wearing of gloves, gowns, eye protection, N95 masks, practising good personal hygiene, and self‐monitoring for early disease symptoms and early treatment. Although the implementation of such measures on a massive scale was initially challenging, most health systems and HCWs eventually coped. However, sustaining such extensive preventive measures over prolonged periods was difficult.

For the COVID‐19 outbreak, we have witnessed extensive contact tracing and quarantine measures implemented in major Chinese cities, affecting millions of people. Implementing such measures requires advance planning of suitable locations for quarantine, giving support to patients who are in quarantine and being strict to those who break the laws by enforcing penalties. Health care resources have also been marshalled and mobilized on an unprecedented scale to respond to those who require treatment. However, a current challenge is the worldwide shortage of medical supplies such as PPE and medical equipment such as ventilators, and even test kits for diagnosis.

Advice given for the general public for COVID‐19 has seen a greater emphasis placed on hand washing, personal hygiene (such as not touching the face with contaminated hands),

respiratory hygiene (eg, practising cough etiquette by coughing or sneezing away from others or into the sleeves and wearing masks if feeling unwell), social distancing, avoidance of crowds, travel advice and advice that persons who are well need not wear masks.^25^

Due to the limited supply of masks, the initial general advice has been for masks to be worn by ill patients and people who have close contact or who are looking after ill patients. For the general public, information on types of masks to wear (surgical masks would suffice, rather than N95 masks), the proper way of wearing and disposing of masks in a safe and socially responsible manner has been given.^26^ However, it is important to note that such information continues to evolve. The latest guidelines from Centers for Disease Control and Prevention (CDC) in April 2020^27^ recommend that masks or face coverings should be worn by the public when they go out. One reason for the change in advice is the recognition that there may be asymptomatic cases who might spread infected respiratory droplets to other members of the public if they are not wearing face coverings or masks.

Social distancing has been implemented as part of the efforts to “flatten the curve”. The “curve” refers to the projected number of people who will contract COVID‐19 over a period of time. By implementing community isolation measures, the daily number of disease cases can be kept at a manageable level for medical providers, hence it may help lessen the healthcare burden.^28^

As the situation evolves, some countries are employing more restrictive measures such as travel bans and lockdowns. It was reported that by the end of March 2020, more than 100 countries had instituted either a full or partial lockdown, impacting billions of residents.^29^

## 4 THE DISEASE POSES BOTH A PUBLIC HEALTH AS WELL AS AN OCCUPATIONAL HEALTH THREAT

Severe acute respiratory syndrome was widely viewed as a public health threat but was less appreciated as an occupational disease. Among the occupational groups at risk were HCWs, animal and food preparation handlers, transport workers (ranging from flight attendants to taxi drivers), and laboratory researchers working with the SARS‐CoV. For example, more than a third of the early cases of SARS (pre‐February 2003) occurred in persons who handled, killed, or sold food animals, or in those who prepared or served food. Thus, in addition to public health measures, an appropriate occupational health response is also necessary.^10^

In the current COVID‐19 outbreak, diverse occupational groups are recognized to be at risk. For example, in Singapore, 68% of the first 25 locally transmitted cases were probably related to occupational exposure.^30^ The workers who are in the hospitality, retail, food and beverage industry who served infected tourists, transport workers, multinational company workers who attended an international meeting, a domestic worker and even a security officer who served quarantine orders were all at risk. These occupations were not covered by the occupational health legislation of Singapore in 2003, which was the Factories Act. However, in 2020, the Workplace Safety and Health Act in Singapore applies to many of these workers. Thus, if and when the disease is officially recognized as an occupational disease, these workers (and not only factory workers) will be covered by the health and safety law.

A major concern of many workers is the fear of job losses or loss of income. This is apparent from the economic impact of COVID‐19 where in many countries, non‐essential services have been halted, and many people stay at home and avoid going out for shopping or entertainment. Self‐employed workers, workers in a gig economy, and those working in entertainment, hospitality, tourism and travel sectors, to name a few, will be threatened with loss of income and job losses. In order to manage the economic fallout, many governments have provided stimulus packages to assist such groups.

Companies and occupational health departments may also play a role in the national and international pandemic response and in managing such concerns among workers under their care.^31^

## 5 EMERGING INFECTIOUS DISEASES WILL POSE A CONTINUING THREAT TO THE WORLD

Since August 2003, after the containment of SARS, new diseases have periodically surfaced to confront the world on a regular basis. These include the appearance of viruses eg, MERS‐CoV, various influenza viruses eg, H1N1 (Swine flu), H5N1 (Avian influenza), H7N9, Zika virus, and the deadly Ebola virus. Such ongoing and sporadic events remind us that it is a certainty that emerging infections will continue to remain as real threats to our world, and we should be ever vigilant and ready to respond.

## References

[1] WHO Statement Regarding Cluster of Pneumonia Cases in Wuhan, China . 2020 https://www.who.int/china/news/detail/09-01-2020-who-statement-regarding-cluster-of-pneumonia-cases-in-wuhan-china. Accessed January 9, 2020.

[2] WHO Statement on the second meeting of the International Health Regulations (2005) Emergency Committee regarding the outbreak of novel coronavirus (2019‐nCoV). 2020 https://www.who.int/news-room/detail/30-01-2020-statement-on-the-second-meeting-of-the-international-health-regulations-(2005)-emergency-committee-regarding-the-outbreak-of-novel-coronavirus-(2019-ncov). Published January 30, 2020. Accessed February 18, 2020.

[3] WHO. Coronavirus disease 2019 (COVID‐19) situation report – 76. 2020 https://www.who.int/docs/default-source/coronaviruse/situation-reports/20200405-sitrep-76-covid-19.pdf?sfvrsn=6ecf0977_2. Published April 5, 2020. Accessed April 6, 2020.

[4] Mahase E . Coronavirus: covid‐19 has killed more people than SARS and MERS combined, despite lower case fatality rate. BMJ. 2020;m641. Published online February 18, 2020. Accessed April 6, 2020. 32071063 10.1136/bmj.m641

[5] Li Q , Guan X , Wu P , et al. Early transmission dynamics in Wuhan, China, of novel coronavirus–infected pneumonia. N Engl J Med. 2020;382(13):1199–1207. 31995857 10.1056/NEJMoa2001316 PMC7121484

[6] Rothe C , Schunk M , Sothmann P , et al. Transmission of 2019‐nCoV infection from an asymptomatic contact in Germany. N Engl J Med. 2020;382(10):970–971. 32003551 10.1056/NEJMc2001468 PMC7120970

[7] Report of the WHO‐China joint mission on coronavirus disease 2019 (COVID‐19). https://www.who.int/publications-detail/report-of-the-who-china-joint-mission-on-coronavirus-disease-2019-(covid-19) Published 28 February 2020. Accessed April 6, 2020.

[8] Swerdlow DL , Finelli L . Preparation for possible sustained transmission of 2019 novel coronavirus: Lessons from previous epidemics. JAMA. 2020;323(12):1129. Published online February 11, 2020. Accessed April 6, 2020. 32207807 10.1001/jama.2020.1960

[9] Koh D , Sng J . Lessons from the past: Perspectives on severe acute respiratory syndrome. Asia Pac J Pub Health. 2010;22(3):132S‐136S. 20566545 10.1177/1010539510373010

[10] Koh D , Lim M‐K , Ong C‐N , Chia S‐E . Occupational health response to SARS. Emerg Infect Dis. 2005;11:167‐168. 10.3201/eid1101.040637 PMC3294325 15714660

[11] Secon H . Nearly 3,400 Chinese healthcare workers have gotten the coronavirus and 13 have died. 2020 https://www.businessinsider.com/healthcare-workers-getting-coronavirus-500-infected-2020-2 Published 5 March 2020. Accessed April 7, 2020.

[12] Wang D , Hu B , Hu C , et al, Clinical characteristics of 138 hospitalized patients with 2019 novel coronavirus‐infected pneumonia in Wuhan, China. JAMA. Published online February 7, 2020. Accessed April 6, 2020. 10.1001/jama.2020.1585 PMC7042881 32031570

[13] Gan WH , Lim JW , Koh D . Preventing intra‐hospital infection and transmission of COVID‐19 in healthcare workers. Saf Health Work. 2020. Accessed April 6, 2020. 10.1016/j.shaw.2020.03.001 PMC7102575 32292622

[14] WHO . Coronavirus disease 2019 (COVID‐19) situation report – 29. Coronavirus disease (COVID‐19) outbreak: Rights, roles and responsibilities of health care workers, including key considerations for occupational safety and health. 2020 https://www.who.int/docs/default-source/coronaviruse/who-rights-roles-respon-hw-covid-19.pdf?sfvrsn=bcabd401_0. Published February 18, 2020. Accessed April 6, 2020.

[15] WHO . Coronavirus disease (COVID‐19) technical guidance: Infection prevention and control / WASH. 2020 https://www.who.int/emergencies/diseases/novel-coronavirus-2019/technical-guidance/infection-prevention-and-control. Accessed April 7, 2020.

[16] Ranney ML , Griffeth V , Jha AK . Critical supply shortages—The need for ventilators and personal protective equipment during the Covid‐19 pandemic. NEJM. 2020. Published March 25, 2020. Accessed April 7, 2020. 10.1056/NEJMp2006141 32212516

[17] Godlee F . Protect our healthcare workers. BMJ. 2020;369:2020. Published April 2, 2020. Accessed April 7, 2020.

[18] Maunder R . The experience of the 2003 SARS outbreak as a traumatic stress among frontline healthcare workers in Toronto: lessons learnt. Philos Trans R Soc Lond B Biol Sci. 2004;359:1117‐1125. 15306398 10.1098/rstb.2004.1483 PMC1693388

[19] Koh D , Lim MK , Chia SE , et al, Risk perception and impact of Severe Acute Respiratory Syndrome (SARS) on work and personal lives of healthcare workers in Singapore: what can we learn? Med Care. 2005;43(7):676‐682. 15970782 10.1097/01.mlr.0000167181.36730.cc

[20] Xiang YT , Yang Y , Li W , et al, Timely mental health care for the 2019 novel coronavirus outbreak is urgently needed. Lancet Psychiatry. 2020. Published Online February 4, 2020. Accessed April 6, 2020. 10.1016/S2215-0366(20)30046-8 PMC7128153 32032543

[21] CDC . Reducing Stigma. 2020 https://www.cdc.gov/coronavirus/2019-ncov/daily-life-coping/reducing-stigma.html?CDC_AA_refVal=https%253A%252F%252Fwww.cdc.gov%252Fcoronavirus%252F2019-ncov%252Fsymptoms-testing%252Freducing-stigma.html. Accessed April 7, 2020.

[22] Greenberg N , Docherty M , Gnanapragasam S , Wessely S . Managing mental health challenges faced by healthcare workers during COVID‐19 pandemic. BMJ. 2020;368:m1211. Published March 26, 2020. Accessed April 7, 2020. 32217624 10.1136/bmj.m1211

[23] Rosenbaum L . Facing Covid‐19 in Italy—ethics, logistics, and therapeutics on the epidemic’s front line. N Engl J Med. 2020. Published March 18, 2020. Accessed April 6, 2020. 10.1056/NEJMp2005492 32187459

[24] Truog RD , Mitchell C , Daley GQ . The toughest triage: allocating ventilators in a pandemic. N Engl J Med. 2020. Published March 23, 2020. Accessed April 7, 2020. 10.1056/NEJMp2005689 32202721

[25] WHO . Coronavirus disease (COVID‐19) advice for the public. 2020; https://www.who.int/emergencies/diseases/novel-coronavirus-2019/advice-for-public. Accessed April 7, 2020

[26] WHO . Q&A on coronaviruses. 2020; https://www.who.int/news-room/q-a-detail/q-a-coronaviruses. Accessed April 7, 2020.

[27] CDC . Use of cloth face coverings to help slow the spread of COVID‐19. 2020; https://www.cdc.gov/coronavirus/2019-ncov/prevent-getting-sick/diy-cloth-face-coverings.html. Accessed April 7, 2020.

[28] Stevens H . Why outbreaks like coronavirus spread exponentially, and how to “flatten the curve”. https://www.washingtonpost.com/graphics/2020/world/corona-simulator/. Published on March 14, 2020. Accessed April 7, 2020.

[29] BBC . Coronavirus: a visual guide to the world in lockdown. 2020; https://www.bbc.com/news/world-52103747. Accessed April 7, 2020.

[30] Koh D . Occupational risks for COVID‐19. Occup Med, 2020;70:3–5. Published February 28. 10.1093/occmed/kqaa036 PMC7107962 32107548

[31] Fadel M , Salomon J , Descatha A . Coronavirus outbreak: the role of companies in preparedness and responses. Lancet Pub Health. 2020;5(4):e193. Epub 2020 Feb 28. 32119831 10.1016/S2468-2667(20)30051-7 PMC7129529

